# Asthma and COPD Beyond the Airways: Exploring Neurocognitive Links Through NF-κB Subunits c-Rel and p65

**DOI:** 10.3390/ijms26115217

**Published:** 2025-05-29

**Authors:** Magdalena Figat, Aleksandra Wisniewska, Jacek Plichta, Joanna Milkowska-Dymanowska, Sebastian Majewski, Michal S. Karbownik, Piotr Kuna, Michal G. Panek

**Affiliations:** 1 Department of Internal Medicine, Asthma and Allergy, Medical University of Lodz, 90-153 Lodz, Poland; magdalena.figat@gmail.com (M.F.); jacek.plichta@umed.lodz.pl (J.P.);; 2 Department of Clinical Pharmacology, I^st^ Chair of Internal Medicine, Medical University of Lodz, 90-153 Lodz, Poland; aleksandra.wisniewska@umed.lodz.pl; 3 Department of Pneumology, Medical University of Lodz, 90-153 Lodz, Polandsebastian.majewski@umed.lodz.pl (S.M.); 4 Department of Pharmacology and Toxicology, Medical University of Lodz, 90-752 Lodz, Poland; michal.karbownik@umed.lodz.pl

**Keywords:** asthma, COPD, cognitive impairment, c-Rel, p65, NF-κB, neuroinflammation, chronic inflammation

## Abstract

The evolving understanding of asthma and COPD pathomechanisms led to this study examining chronic obstructive lung diseases’ impact on cognitive decline—a growing concern in aging populations. We explored whether subunits of key inflammatory regulators NF-κB, c-Rel (neuroprotective), and p65 (neurodegenerative), are linked to cognitive impairment. A pilot study with an explorative design across three groups (asthma, COPD, and control) included 78 patients. Participants underwent assessments via 16 questionnaires (covering demographics, quality of life, disease control, and cognitive and psychiatric evaluations), spirometry, and blood sampling to measure c-Rel and p65 mRNA expression. While both c-Rel and p65 are NF-κB subunits, their expression levels differ independently. Median c-Rel expression was highest, and p65 lowest, in the group with the best cognitive function (control). The most notable correlations for both markers with PKA, CREB, MMSE, and HAM-D were in COPD. The significant association between p65 and the Clock-Drawing Test, without a corresponding link to MMSE, may indicate that a future correlation between p65 and cognitive decline, as assessed by CDT, is likely to emerge.

## 1. Introduction

Monitoring lung function, exacerbation frequency, and quality of life remains central to the management of chronic obstructive lung diseases (asthma and COPD). However, current guidelines place limited emphasis on the comprehensive assessment of other organ systems that may also be affected.

According to data from a nationally representative sample in the United States, the two-year incidence of Alzheimer’s disease (AD) is 1.1% in individuals without asthma and 1.4% in those with asthma [1]. Even the most recent report from the Global Initiative for Chronic Obstructive Lung Disease (GOLD) states that 32% of patients with chronic obstructive pulmonary disease (COPD) are diagnosed with cognitive impairment, with estimates suggesting that the true prevalence may be as high as 56% [2]. For comparison, the prevalence of cognitive impairment in the general population of high-income countries is estimated at 5–10% [3].

Multivariable models suggest that local inflammation, such as that seen in asthma, may be a modifiable risk factor for dementia [4]. The progression of inflammation into the central nervous system (CNS), leading to neurodegeneration and synaptic damage, has been supported by elevated concentrations of neurogranin and α-synuclein in cerebrospinal fluid [4,5]. At the same time, chronic obstructive lung disease not only constitutes a risk factor for cognitive decline, but also limits engagement in preventive strategies—such as regular physical activity—that are known to protect against neurodegeneration.

Physical activity plays a critical role in mitigating physiological brain aging by helping to preserve brain volume across the lifespan [6]. Yet, chronic obstructive lung diseases inherently restrict physical activity. In asthma, exertion may even trigger acute exacerbations due to hyperventilation [7], further compounding the risk of reduced mobility.

Moreover, aging itself is associated with increased low-grade systemic inflammation, which compromises the integrity of the blood–brain barrier (BBB) [8]. These overlapping factors—limited physical activity, systemic inflammation, and BBB disruption—may contribute to neurocognitive decline in patients with chronic obstructive lung diseases.

A brief examination of the pathophysiology of two most common chronic obstructive lung disease reveals inflammation and hypoxia as primary factors. Both conditions are chronic and obstructive but truly heterogenous. Exposure to the allergens or hyperventilation, which alters osmolality, leads to the excessive production of Immunglobuline E (IgE), resulting in the development of asthma [7]. Inflammation in the lungs also involves mast cells sensitized by IgE [7]. The secretion of interleukin (IL-) 6 regulates CD4+ T-lymphocytes [9], which are responsible for activation of Th2 cytokins such as IL-4, IL-5, IL-9, and IL-13 [10]. IL-4 and IL-13 primarily focus on further IgE production [10]. IL-9 induces the mast cells degranulation, increases mRNA expression of the α chain of the high-affinity IgE receptor, and supports local IgE production in non-atopic phenotypes and generalized IgE production in atopic phenotypes [10]. IL-5 stimulates eosinophil production in bone marrow [7], which, directed by the eotaxin and eosinophil chemotactic cytokines, migrates to the airways [10]. Under the influence of transforming growth factor ß (TGF-ß), this can lead to airway remodeling and exacerbations [7]. Severe asthma might be driven by tumor nekrosis factor α (TNF-α) and IL-1ß [7]. Concurrently, this results in the activation the nuclear factor kappa-light-chain-enhancer of activated B cells (NF-κB), which amplifies and perpetuates the inflammatory mechanism [11].

Exposer to smoking and biomass activates the lung epithelial cells and macrophages [12], leading to an increased accumulation of neutrophils through the granulocyte colony-stimulating factor (G-CSF) and chemoatrractant CXCR2 [7,12]. Neutrophils secrete serine protease in lungs, such as matrix metalloproteinase (MMP)-8 and MMP-9, neutrophil elastase, cathepsin G, and proteinase 3 [7]. The latter three stimulate the submucosal glands and goblet cells, causing mucus hypersecretion [13]. Neutrophil elastase also triggers eosinophil degranulation [14]. All these proteases collectively drive the alveolar destruction typical for COPD [7]. Simultaneously, the NF-κB/MAPK signaling pathway may be directly induced by smoking or oxidative stress [12], further enhancing the activation of neutrophils and macrophages.

An overview of the pathomechanism in each disease reveals that IL-5R expression is not limited to eosinophils. It can also be found on airway epithelial cells [15], neutrophils [16], and mast cells [17]. This expression can drive inflammation independent of the Th2 pathway, a mechanism increasingly associated with COPD, as well as asthma. Additionally, up to 40% of COPD cases exhibit an eosinophilic phenotype [18,19], characterized by elevated eosinophil counts in both peripheral blood und sputum, indicative of Th2-dependent inflammation—typically more characteristic of asthma.

Regardless of the dominant cell type in both diseases, inflammation is amplified by NF-κB. The upregulation of this factor activates its individual domains, including c-Rel, p65, p50, and p52. The ultimate effect can be neuroprotective or deleterious, depending on the activated domains [20]. The p65 subunit is strongly correlated with cholinergic degeneration, as seen in comparisons with Alzheimer’s disease or multiple sclerosis patients [20]. On the contrary, the activation of c-Rel promotes neuroprotection [20].

Both chronic obstructive lung diseases (asthma and COPD) lead to insufficient oxygen replenishment, which directly constitutes a risk factor for dementia development [21,22]. Hypoxia dysregulates the expression of HIF-1α and may contribute to the degeneration of affected tissue [23]. The further progression of hypoxia, including its effect on the cerebral system, has been demonstrated in a murine model to begin with neuronal dysfunction and cognitive impairment, potentially ending with cell death [24]. In the ongoing study of Alzheimer diseases pathogenesis, hypoxia has also been identified as a factor that supports the formation of pathological proteins such as amyloid β and tau, as well as neuronal degeneration [25]. Hypoxia exacerbates the dysfunction of the BBB, facilitating the smoother transport of inflammatory cytokines [25].

The intensified accumulation of eosinophil in asthma and neutrophils in COPD increased the generation of reactive oxygen species (ROS) [26,27], leading to an imbalance known as oxidative stress. Consequently, Th2 immune response may be altered, resulting in the upregulation of NF-κB [28]. Therefore, the expression of inflammatory and immune genes mediated through NF-κB is increased. This group of genes includes enzymes such as the inducible form of nitric oxide synthase (producing NO) and inducible cyclooxygenase (producing prostaglandins), as well as adhesion molecules like E-selectin, vascular cell adhesion molecule-1 (VCAM-1), and intercellular adhesion molecule-1 (ICAM-1), which play crucial roles in the recruitment of inflammatory cells. Among the products of these groups are proinflammatory cytokines such as IL-1β and TNF-α, completing a positive regulatory loop that can amplify and perpetuate the inflammatory response [11].

The Coop Center Longitudinal Study, with cohorts of patients with chronic obstructive lungs diseases such as asthma, reported 78% increased risk of cognitive impairment linked to asthma [29]. Additionally, lower cognitive function is observed in patients with an uncontrolled course of the disease [30]. Furthermore, longer disease duration and lower lung function are more strongly related to cognitive detriment [31]. A meta-analysis of similar original research concluded that intermittent cerebral hypoxia might be a reason for cognitive disturbances associated with asthma [32]. The Mayo Clinic published data from a prospective population-based study with COPD patients, revealing an 83% increased risk of mild cognitive impairment [33]. Following a systematic review and meta-analysis of observational studies, the prevalence of any cognitive impairment in COPD is 32% [34]. Based on the presented analysis of molecular pathways and clinical data, the hypothesis of a positive correlation between c-Rel expression and better cognitive function—as well as a positive association between p65 expression and poorer cognitive performance—appears well supported. This raises the question of why no studies have directly considered any molecular marker to follow changes in cognitive functions in chronic obstructive lungs diseases. Our project appears to be the first study in chronic obstructive lung disease, encompassing both asthma and COPD, to investigate the linkage between cognitive performance and the mRNA expression level for two NF-κB subunits: c-Rel and p65.

The aims of this study were as follows:

1. To examine the c-Rel and p65 mRNA expression in each group.
2. To identify any relationships between the results of neuropsychiatric evaluation and c-Rel or p65 mRNA expression.

## 2. Results

### 2.1. Baseline Characteristics

A total of 78 patients were recruited and divided into three groups. The asthma group included 26 patients (11 female), with a mean age of 55 ± 16.45 years. The COPD group initially included 28 patients; however, one patient was excluded due to screening failure, resulting in 27 participants (13 female) with a median age of 66 ± 7.61 years. For the control group (CG), 32 individuals were initially recruited. Following age-matching to align with the asthma group, the control group consisted of 25 participants (14 female) with a mean age of 53 ± 14.47 years.

Descriptive analysis between groups revealed notable age differences, particularly in the asthma–COPD and COPD–CG comparisons. Significant variations were also observed in educational level, household size, and residence. Given the pathomechanisms and typical age of first manifestations in each condition, these results were accepted and further verified through adjustments using relevant covariates [35,36,37]. The covariates included age, gender, educational level, residence, mental activity, and potential risk factors for cerebral damage such as prior anesthesia [38], nicotine consumes, obesity, and systemic steroid therapy. Detailed demographic data are available in the supplementary materials for “Potential Association Between Obstructive Lung Diseases and Cognitive Decline” [39]. This study presents a partial secondary analysis of the data originally published there.

The presence of a reported discrepancy in cognitive function between the control group and patients with obstructive lung diseases (without distinguishing between asthma and COPD) in the aforementioned article [39] led to the development of a new research hypothesis and a search for potential molecular markers of cognitive decline.

**Figure 1:**
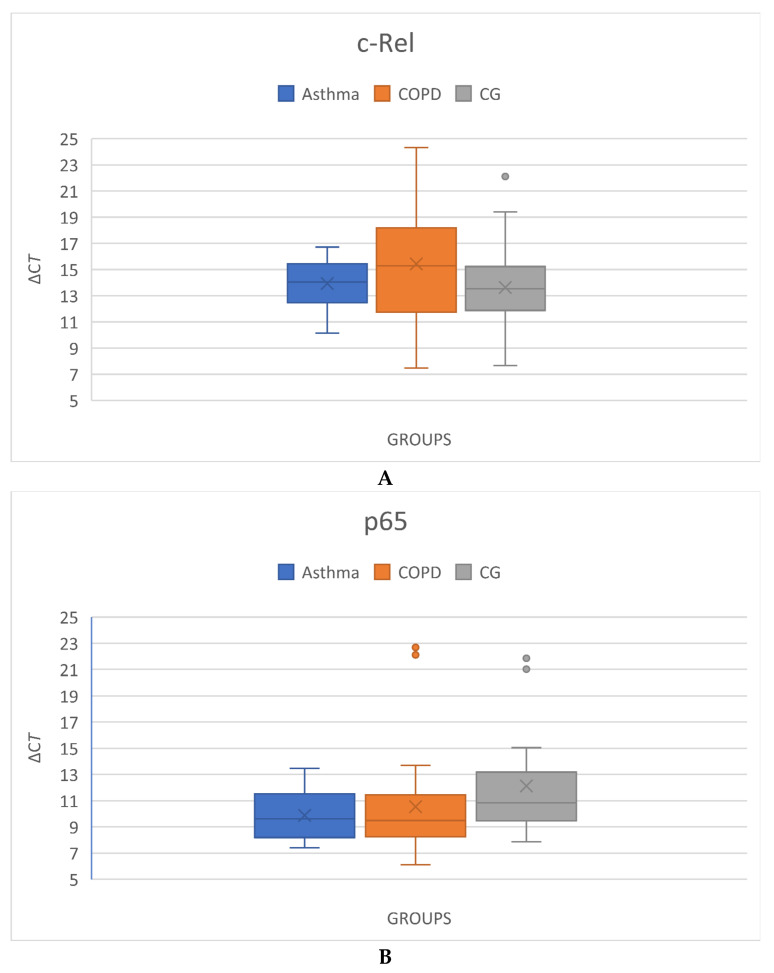
The average mRNA expression values of c-Rel (**A**) and p65 (**B**) for each group. The upper points represent the most extreme outlier measurements in both groups. The cross indicates the mean value.

The mRNA expression levels for both genes showed a normal distribution in the study population (c-Rel: *p* = 0.006, p65: *p* = 0.000). The mean expression level of c-Rel was highest in the control group (CG) (−ΔC_T_ = −13.624 ± 3.249) and lowest in the COPD group (−ΔC_T_ = −15.426), with a broader standard deviation (SD = 4.44). The asthma group had a lower mean expression than the CG (−ΔC_T_ = −13.932 ± 1.939) but higher than the COPD group, with a narrower SD, indicating greater consistency (Figure 1A). When comparing mRNA expression levels across groups, the standard error (SE) was lowest in the COPD group (asthma and CG: 0.680; COPD: 0.655). The mean mRNA expression of p65 followed a different pattern, with the CG showing the lowest values and the asthma group the highest (−ΔC_T CG_ = −12.122 ± 3.930; −ΔC_T COPD_ = −10.537 ± 3.917; −ΔC_T asthma_ = −9.873 ± 1.815) (Figure 1B). The SE values for each group demonstrated minimal differences (CG: 0.680; COPD: 263 0.655; asthma: 0.695).

### 2.2. Relationship Between Neuropsychiatric Evaluation Results and c-Rel/p65 mRNA Expression

Adjustment for the specified covariates had minimal effect on the median mRNA expression in the asthma group for both gene domains (Table 1). However, the SE increased across all groups, along with a greater divergence between them. Following the neuropsychiatric assessment and prior evaluation of molecular factors, we investigated potential associations with PKA, CREB, and c-Rel and p65 expression (Table 2). In the study population, which includes all asthma and COPD patients, and the control group (CG), notable relationships were observed between c-Rel and PKA (β = 0.241, *p* = 0.035), CREB (β = 0.256, *p* = 0.025), MMSE (β = 0.288, *p* = 0.011), and ATMS (β = 0.273, *p* = 0.016) (B-H-corrected significance level 0.047). All these associations remained significant after adjusting for covariates, except from that for ATMS, which did not survive B-H correction. In subgroup analyses, the significance of these associations was mirrored only for MMSE in COPD patients (β = 0.497, *p* = 0.008) (B-H-corrected significance level 0.0093). In the asthma group, the association maintained positive (β = 0.088) but did not reach statistical significance (B-H-corrected significance level 0.00937, *p* = 0.675). Conversely, in the CG, the β value for MMSE and c-Rel association was negative (−0.162), also without statistical significance (B-H-corrected significance level 0.00937, *p* = 0.440). After adjustment, the correlation between c-Rel and MMSE in COPD lost significance. Further analysis in COPD revealed an additional association between c-Rel and both PKA (β = 0.573, *p* = 0.047) and CREB (β = 0.579, *p* = 0.022), but they did not withstand the B-H correction, with the corrected significance level of 0.00937.

In the COPD group, the mRNA expression of p65 showed borderline significant correlations. Specifically, a correlation was found with PKA (β = 0.393, *p* = 0.043), with a similar β direction observed in the CG and asthma groups, though these were not statistically significant. A second association was identified with HAM-D (β = −0.398, *p* = 0.040), which was also not significant after B-H correction in the asthma and CG groups. The final correlation was observed with for p65 and CREB in raw data (β = 0.421, *p* = 0.029); however, both the asthma and CG groups exhibited negative β values, and statistically non-significant results (*p* = 0.735). After adjustment, no significant associations with p65 or c-Rel were observed in the COPD group. No p65 correlation survived the B-H correction in COPD (B-H-corrected significance level 0.00937).

Considering the outcomes of the CDT, we noted a significant difference in the mRNA expression of p65 in the total study sample in raw analysis (*p* = 0.035; B-H-corrected significance level 0.0469) (Table 3). Overall, correctly performed CDT was associated with lower p65 expression. A similar trend was observed in the asthma group, although it did not reach statistical significance (*p* = 0.217). Surprisingly COPD appeared to present the opposite results. Additionally, lower c-Rel mRNA expression seems to be associated with improper performance on the CDT and did not show any significant correlation with other genes across the groups. The control group could not be included in the analysis, as all participants performed the test correctly. In the adjusted data, only the mRNA expression values of p65 for the entire study population approached significance (*p* = 0.056).

Subsequently, a logistic regression analysis was performed to examine the relationship between CDT results and mRNA expression levels for c-Rel and p65. c-Rel did not demonstrate any significant correlation; therefore, no adjustments were made. Notably, significant results were observed for p65 in the study population. The probability of correctly performed CDT decreased by 25% with each unit increase in −ΔC_T_ for p65. No significance was reported following adjustments.

## 3. Discussion

Although c-Rel and p65 are both subunits of NF-κB, their expression levels are independent and distinctly different. The median values of mRNA expression for c-Rel and p65 support theoretical assumptions: c-Rel exhibited the highest levels, while p65 showed the lowest levels in the group with the best cognitive function (CG), as indicated by superior neurocognitive and neuropsychiatric assessments, regarding their respective neuroprotective and neurodegenerative roles. A more detailed analysis within each group revealed potential correlations with neuropsychiatric assessments.

The second important observation in our study is that both c-Rel and p65 exhibited the most significant correlations with PKA, CREB, MMSE, and HAM-D, mostly in the COPD group. Specifically, higher c-Rel expression correlated with better results on the MMSE and AMTS; both questionnaires are structured such that higher scores indicate better cognitive status in patients. However, the correlation between AMTS and c-Rel was rejected by B-H correlation. Also, the significant association between c-Rel and MMSE observed in COPD was not confirmed after covariate adjustment, but, for raw data, survived in B-H correction. Furthermore, additional analyses of group correlations did not yield significant results. This indicates that MMSE results might not be directly used to predict c-Rel mRNA expression levels in COPD patients. Further research is needed.

In the case of p65, some correlations were observed, but only within the COPD group. The relationship with CREB may relate to findings from previous analyses [39], which indicated a significant correlation between CREB mRNA expression and poorer cognitive outcomes in the COPD group. Additionally, an interesting association was detected between p65 and HAM-D. The negative β-value indicates that an increasing HAM-D score is associated with a decreasing level of p65 mRNA expression. A higher HAM-D score reflects a more depressive state in patients, which could suggest lower p65 expression. This result somewhat contradicts the theoretical pathways presented in the introduction. Interestingly, the finding appeared insignificant following B-H correction.

The initial hypothesis can be further supported by analyzing the correlations with the CDT. Higher levels of c-Rel mRNA expression are associated with correctly performed CDT across all groups, while higher levels of p65 mRNA expression are linked to incorrectly performed CDT. This supports the hypothesis that p65 functions in a neurodegenerative capacity, a finding that holds true for both the study population and the asthma group. In contrast, the COPD group exhibited an association in the opposite direction. A possible explanation for this discrepancy may be the presence of coexisting conditions in severe COPD, such as hypoxia and carbon dioxide accumulation.

The Clock-Drawing Test is one of the oldest screening tools for dementia and is considered more sensitive than the MMSE [40,41]. This may explain why the association between p65 and the CDT does not correlate with the MMSE results. It is highly probable that corresponding correlations may be identified in follow-up studies conducted over several years.

Reviewing the generated observations, they appear to align with findings in basic science. The expression of p65 is primarily reported in pathologically activated cells within the canonical pathway, which mediates neuroinflammation and leads to apoptosis in the CNS [20]. Thus, inactivation of this gene may confer protective effects on the CNS. However, the knockout of p65 in mouse models results in the development of a defective immune system and TNF-dependent liver apoptosis, often leading to lethality during the embryonic phase [42]. In contrast, the knockout of c-Rel is associated with more moderate consequences. Subjects with c-Rel knockout can develop normally, but exhibit impaired T- and B-cell activation, defective macrophage function [42], and an increased systemic inflammatory response due to significant potentiation of several TNF-α-induced mediators of inflammation [43].

p65 is a key protein involved in inflammation and the regulation of IL-1β, IL-6, and TNF-α in the hippocampus [44]. It is typically upregulated in asthma and downregulated by montelukast [44], which corresponds with our findings; indeed, our data show that p65 is significantly upregulated in the asthma group. The downregulating effect of montelukast is not evident in our results, likely due to the recent GINA therapy recommendations that classify it as an optional rather than a foundational medication, particularly for children [45]. In contrast, c-Rel is responsible for suppressing TNF-α-induced RelA-dependent transactivation of its promoters, including Ig-κB, IP-10, and A20 [43]. Mathematical analyses of memory-associated genes containing c-Rel elements in their upstream DNA regions within the hippocampus identified that five out of seven are associated with regulatory factors such as CREB [46]. This finding supports the role of c-Rel in regulating transcription during memory consolidation [46]. A similar correlation was also observed in our data (Table 2). The exact mechanism of action in long-term synaptic plasticity and memory formation may depend on the type and duration of the stimulus, as well as specific signaling pathways [46]. Studies of p65-deficient mice, which exhibit notable learning deficits, highlight the complexity of synaptic activation and regulation during synaptic plasticity and memory formation [46].

Both factors play crucial roles in maintaining the balance between anti-inflammatory and inflammatory, as well as apoptotic and anti-apoptotic, processes. c-Rel, as a homodimer, appears to be more stable than p65 in this form [46]. Conversely, the p65/p50 heterodimer is more abundant than the c-Rel/p50 complex [46]. This abundance can vary across different cell types, allowing for specificity in NF-κB pathway signaling related to specific cell functions. A prime example of this is in neurons, where growth and survival are influenced and regulated by p65 expression [46,47].

The activation of the p65/p50 heterodimer occurs through pathological stimuli via the canonical pathway, which is faster than the non-canonical pathway [48]. This process is characterized by the predominant phosphorylation of the Rel homology dimers by protein kinase A (PKA). The catalytic subunit of PKA must be activated by the IKK complex, which induces a structural change in p65, facilitating its interaction with the cAMP response element-binding protein (CREB) and enhancing transcriptional activity [48]. Phosphorylation is one of the several modifications that can influence p65 activity. For instance, the activation of p38 mitogen-activated protein kinase (MAPK) can be used to inhibit transcriptional activity [48,49]. Manipulating specific transcription factors can lead to both pro-inflammatory and anti-inflammatory effects, making it crucial to understand the consequences of these actions in detail. This study demonstrates potential associations with neurocognitive functions.

Overall, this study is not without limitations. Due to its pilot design and exploratory nature, the sample size of the groups was limited, which resulted in a few significant findings. However, the composition of the patient groups aligns with the latest reports on the pathomechanisms underlying obstructive lung diseases such as asthma and COPD, providing an innovative overview and comparison of these two conditions within a single study. Due to the exploratory nature of the study, the false discovery rate was slightly relaxed and set at 0.15. While this may be considered as a limitation, it also increased the sensitivity of the statistical analysis, allowing for the identification of potentially meaningful correlations that may serve as a valuable basis for future research in this field. Furthermore, the molecular analysis correlated with well-established and validated neuropsychiatric tools, enhancing the overall value of this project. Another limitation may be the use of peripheral blood for molecular gene expression analysis. While this approach is standard in clinical studies, it must be acknowledged that it does not directly reflect processes within the CNS. However, NF-κB is known to amplify systemic inflammation [20], which can increase the permeability of the BBB [8]. Based on the “outside-in” mechanism, it is plausible that systemic inflammation may impact the CNS [50,51,52]. This hypothesis suggests that inflammation is not confined to the lungs, but may spread to the brain, potentially contributing to neurodegeneration. This risk aligns with data on comorbidities presented in the GOLD Guidelines [53]. To resolve—or at least better define—the discrepancies between peripheral blood and CNS-specific activity, future studies could expand molecular gene expression analysis to include cerebrospinal fluid. Future expansions and the incorporation of follow-up examinations could lead to compelling conclusions.

## 4. Materials and Methods

### 4.1. Bioethics Committee

The study was approved by the Bioethics Committee, resolution number: RNN/287/19/KE (13 June 2019).

### 4.2. Characteristics of the Groups at the Baseline

Two patient groups were established from University Clinical Hospital No. 1 N. Barlicki. Recruitment was conducted in both outpatient and inpatient clinics across two departments: The Department of Internal Medicine, Asthma and Allergy and the Department of Pneumonology. The asthma group consisted of patients with confirmed diagnosis of severe asthma based on GINA Guidelines [45]. For the COPD group, patients were required to have COPD classified as GOLD 2 or 3, B or E, according to GOLD Guidelines [2,53]. A third group comprised healthy volunteers selected from general population. None of the participants had been diagnosed with, or were undergoing diagnostic processes for, neurodegenerative diseases such as dementia, Alzheimer’s Disease, Parkinson’s Syndrome, mild cognitive impairment, multiple sclerosis, or similar conditions.

No power calculation was performed prior to recruitment, and the group sizes were determined based on available financial resources. This project was conducted as a pilot to generate preliminary findings, and as such, the results may be underpowered.

### 4.3. Eligibility Criteria

To participate in the study, each patient was required to sign an informed consent form, be at least 18 years old, and suffer from one of the chronic obstructive lung diseases. Asthma patients with confirmed diagnoses according to the GINA 2023 [45], and COPD patients according to GOLD Standards 2024 [53], underwent baseline spirometry, assessed in accordance with ERS Standards [54].

For control group participants, the same criteria of being at least 18 years old and providing informed consent applied. Their lung function was evaluated at baseline using spirometry, following the same ERS technical standards [54], to exclude any potential lung disorders.

In all three groups, participants were required to have no neurodegenerative conditions or ongoing diagnostic process related such conditions. All participants had to agree to blood sampling and complete the required questionnaires.

Exclusion criteria included being under 18 years of age, not signing the informed consent, refusal to undergo spirometry or blood sampling, the presence of other lung diseases, and any neurodegenerative disease or suspicion thereof.

### 4.4. Examination Procedure

During the examination, as previously mentioned, spirometry was performed according to ERS standards [54]. Non-fasting venous blood samples were collected, followed by the administration of a comprehensive set of 16 questionnaires. These questionnaires can be categorized into four main groups. The first group includes (1) demographic data, and general questionaries assessing the quality of life, such as (2) the Index of Independence of Daily Living (ADL) [55,56], (3) the Lawton Instrumental Activities of Daily Living (IADL) [57], and (4) the 36-item Short Form Healthy Survey (SF-36) [58,59]. In this study, the interpretation of SF-36 was based on the methodology presented in the “Methodology of quality of life assessment” [60]. The SF-36 assesses eight domains, divided into two components: mental and physical. Median values of these components are reported. The more commonly used method, known as Taft’s Hypothesis, assumes a reciprocal relationship between the physical and mental domains, where a clinical change would be reflected by decreasing in one component and an increase in the other [58,61]. However, due to its easier implementation in statistical analysis, the first method was chosen.

The second part of the assessment consisted of disease-specific questionnaires designed to evaluate disease control. For the asthma group, the following were used: (5) the Asthma Control Test TM (ACT TM) [62], (6) the Asthma Control Questionnaire (ACQ) [63], and (7) the standardized Asthma Quality of Life Questionnaire (AQLQ(S)) [64], all in accordance with current GINA Guidelines. In the COPD group, the following tests recommended by GOLD standards were administered: (8) the COPD Assessment Test (CAT) [65,66], (9) the modified Medical Research Council dyspnea scale (mMRC) [67], and (10) the St. George’s Respiratory Questionnaire for COPD patients (SGRQ-174 C) [68,69].

In the third part of the study, a direct assessment of cognitive function was conducted. The following standard neurological tools were used: (11) the Mini-Mental State Examination (MMSE), which assesses cognitive aspects such as mental trucking, expressive and receptive language, visual construction, and immediate and delayed free verbal recall, calculation, and temporal and spatial orientation [70,71,72]. Another widely recognized cognitive test employed was (12) the Clock-Drawing Test (CDT), a cognitive screening tool used for early detection, prediction, or monitoring of cognitive disturbances [73,74]. Additionally, (13) the Abbreviated Mental Test Score (AMTS) was used to further verify cognitive function through short questions and commands [75]. Finally, (14) the Hachinski Ischaemic Score (HIS) was administered to gather the patient’s medical history, including comorbidities and symptoms, to help differentiate between cognitive dysfunctions of senile or vascular origin [76,77,78].

The fourth group of assessments indirectly evaluated cognitive function by performing a psychological evaluation of the participant. (15) The Hamilton Depression Rating Scale, an observer-rated measure, was used to assess depression [79,80]. To achieve a complete picture of patient’s psychological status, a self-rated scale was also employed: (16) the 15-item Geriatric Depression Scale (GDS), which is well known as an effective screening tool for depression [81,82,83].

### 4.5. Bioemical Methods

#### 4.5.1. RNA Extraction

Red and white blood cells were separated using the RBCL cell lysis medium (A&A Biotechnology RBCL medium, REF 213-250, LOT 280222, Gdansk, Poland) according to the manufacturer’s instructions. The isolated white blood cells were used in the RNA extraction, utilizing the A&A Biotechnology Total RNA Mini kit (REF 031-100, A&A Biotechnology, Gdansk, Poland) following the protocol provided by the manufacturer.

#### 4.5.2. RNA Concentration Measurement

The quality of prepared RNA was verified using the Picodrop Spectrophotometer to measure its concentration. This method can detect an RNA concentration ranging from 2 to 3000 μg/mL in 0.5–201 2.0 μL of solution without requiring dilution [84].

#### 4.5.3. Reverse Transcription and qPCR

Reverse transcription was carried out using the ImProm-II Reverse Transcription System (REF A3800, LOT 0000437833, Promega, Madison, WI, USA) according to the provided user guide. This procedure involves the synthesis of complementary DNA, which is then used for subsequent amplification via PCR.

The next step involved performing a qPCR assay using following reagents: TaqMan Universal PCR Master Mix (REF 4304437 LOT 2208187 Applied Biosystem, Waltham, MA, USA); NF-kB c-rel-PrimePCR™ SYBR^®^ Green Assay (REL, Human, BioRad, cat qHsaCID0017568, Hercules, CA, USA); p65-Human RELA/NF-kappa B (p65 qPCR Primer Pair, Sino Biological, cat HP100039, Beijing, China); Applied Biosystems Euk 18S rRNA (20X) REF 4333760F LOT 2003209. According to the manufacturer’s protocol, the plate was read after 45 cycles.

The threshold cycle (C_T_) values for each sample were calculated using Mx-Pro software (V4.10d, Agilent, Santa Clara, CA, USA). For each gene, including c-Rel, p65, and the housekeeping reference gene, 18S, two technical replicates were performed. The difference between the replicates was assessed; if it assumed ≤1 [84], an average was computed. The C_T_ for each gene was then determined using the formula ΔC_T_ = C_T,_ *_GENE_*–C_T,_ *_18S_*. If the difference between the technical replicates exceeded 1, the RT-PCR was repeated, and the same calculation was performed again. The final ΔC_T_ value was derived as the mean of the initial and the repeated RT-PCR measurements. If 45 cycles were insufficient to detect the transcript, the ΔC_T_ value was calculated substituting the C_T,_ *_GENE_* value with 45. The obtained data were then entered into the database and subjected to statistical analysis.

### 4.6. Statistical Analysis

Descriptive statistics included the mean with standard deviation, median with 1st–3rd quartiles, and the number with frequencies for continuous, ordinal, or categorical variables, respectively. General linear model analyses were adopted to examine the associations and group comparisons. For binary outcomes, logistic regression was used. The analyses were additionally adjusted for age, gender, educational level, residence, mental activity, prior anesthesia [38], nicotine consumes, obesity, and systemic steroid therapy. Pairwise deletion was used in the situation of data missingness. To control for the false discovery rate (FDR = 0.15), Benjamini–Hochberg (B-H) correction for multiple testing was used, and *p*-values lower than the B-H-corrected significance level were considered statistically significant. The B-H correction was applied separately for raw and covariate-adjusted models, and independently within each predefined patient subgroup. Analyses were performed with STATISTICA 13.3 software (Statsoft; Tulsa, 231 OK, USA).

## Figures and Tables

**Table 1:** Comparison of median mRNA expression values for c-Rel and p65 in raw and adjusted data.

Group	Datatype	−ΔC*_T c-Rel_*	SE	−ΔC*_T p65_*	SE
Asthma	raw	−13.931	0.680	−9.873	0.694
	adjusted	−13.815	0.786	−9.394	0.821
COPD	raw	−15.426	0.654	−10.537	0.654
	adjusted	−16.340	0.908	−11.445	0.940
CG	raw	−13.624	0.680	−12.121	0.680
	adjusted	−12.969	0.807	−11.644	0.806

SE—standard error, CG—control group, COPD—chronic obstructive lung disease, −ΔC*_T c-Rel_*—delta cycle threshold values for c-Rel, −ΔC*_T p65_*—delta cycle threshold values for p65.

**Table 2:** The relationships between the previously examined mRNA expression levels of CREB and PKA genes, cognitive function test results, psychiatric assessment scores, and the mRNA expression of both NF-κB subunits.

			All *		Asthma		COPD		CG	
			ΔC*_T c-Rel_*	ΔC*_T p65_*	ΔC*_T c-Rel_*	ΔC*_T p65_*	ΔC*_T c-Rel_*	ΔC*_T p65_*	ΔC*_T c-Rel_*	ΔC*_T p65_*
ΔC*_T PKA_*	Raw	β	**0.241**	0.168	−0.059	0.227	0.342	0.393	0.146	0.036
		*p*	**0.035**	0.148	0.780	0.287	0.081	0.043	0.487	0.863
	Adjusted	β	**0.276**	0.222	−0.419	−0.041	0.573	0.468	0.125	−0.265
		*p*	**0.020**	0.073	0.071	0.870	0.047	0.065	0.281	0.298
ΔC*_T CREB_*	Raw	β	**0.256**	0.080	−0.113	−0.021	0.342	0.421	0.383	−0.071
		*p*	**0.025**	0.492	0.589	0.921	0.081	0.029	0.059	0.735
	Adjusted	β	**0.292**	0.093	−0.318	−0.296	0.579	0.434	0.492	−0.166
		*p*	**0.013**	0.455	0.289	0.320	0.022	0.055	0.066	0.543
MMSE	Raw	β	**0.288**	−0.002	0.088	0.122	**0.497**	0.161	−0.162	−0.095
		*p*	**0.011**	0.988	0.675	0.570	**0.008**	0.422	0.440	0.651
	Adjusted	β	**0.215**	0.015	−0.046	0.091	0.166	0.075	0.238	0.036
		*p*	**0.028**	0.883	0.820	0.662	0.510	0.733	0.197	0.842
AMTS	Raw	β	**0.273**	0.054	0.043	0.185	0.351	0.094	0.204	0.202
		*p*	**0.016**	0.641	0.838	0.386	0.073	0.642	0.327	0.334
	Adjusted	β	0.210	0.065	−0.071	0.127	0.062	0.047	0.091	0.066
		*p*	0.042	0.544	0.769	0.569	0.813	0.835	0.732	0.797
HIS	raw	β	−0.183	0.067	−0.082	−0.134	−0.288	−0.022	0.136	0.172
		*p*	0.111	0.567	0.698	0.533	0.145	0.913	0.517	0.412
	adjusted	β	−0.213	0.052	−0.005	−0.180	−0.142	0.096	0.053	0.157
		*p*	0.084	0.687	0.982	0.406	0.605	0.688	0.860	0.590
GDS	raw	β	−0.043	0.051	−0.184	0.255	−0.064	−0.299	0.361	0.016
		*p*	0.713	0.663	0.390	0.241	0.750	0.129	0.076	0.941
	adjusted	β	−0.031	0.019	−0.227	0.479	0.108	−0.384	0.395	−0.133
		*p*	0.775	0.864	0.432	0.074	0.709	0.114	0.091	0.573
HAM-D	raw	β	−0.169	−0.024	−0.025	0.334	−0.328	−0.398	0.139	−0.058
		*p*	0.141	0.839	0.904	0.110	0.095	0.040	0.506	0.784
	adjusted	β	−0.174	−0.051	−0.126	0.414	−0.011	−0.363	0.121	−0.149
		*p*	0.159	0.691	0.684	0.159	0.969	0.165	0.640	0.554

* Asthma + COPD + CG, Bold—statistically significant findings remained after Benjamini–Hochberg correction; the respective corrected significance thresholds were 0.0469 and 0.0281 for the raw and covariate-adjusted analyses in the total sample, and 0.00937 for all subgroup analyses; CG—control group, COPD—chronic obstructive lung disease, ΔC_T Gene_—delta cycle threshold values for named gene, MMSE—Minimal Mental State Examination, AMTS—Abbreviated Mental Test Score, HIS—Hachinski Ischaemic Score, GDS—the 15-item Geriatric Depression Scale, HAM-D—The Hamilton Depression Rating Scale.

**Table 3:** Comparison of mRNA expression levels of c-Rel and p65 according to CDT outcomes (raw and adjusted data).

			−ΔC*_T c-Rel_*	−ΔC*_T p65_*		−ΔC*_T c-Rel_*	−ΔC*_T p65_*
All *	Correct	raw	−14.198	**−11.333**	adjusted	−14.387	−11.461
	Wrong		−14.792	**−9.379**		−14.787	−9.123
	*p*		0.516	**0.035**		0.728	0.056
Asthma	Correct		−13.655	−10.201		−13.597	−10.265
	Wrong		−14.424	−9.217		−14.247	−8.885
	*p*		0.352	0.217		0.494	0.119
COPD	Correct		−15.171	−9.097		−15.544	−9.408
	Wrong		−15.617	−11.233		−15.341	−11.024
	*p*		0.817	0.199		0.935	0.461

* Asthma + COPD + CG; Bold—statistically significant findings remained after Benjamini–Hochberg correction; the respective corrected significance thresholds were 0.0469 and 0.0281 for the raw and covariate-adjusted analyses in the total sample, and 0.00937 for all subgroup analyses; COPD—chronic obstructive lung disease, ΔC_T Gene_—delta cycle threshold values for named gene.

## Data Availability

The original contributions presented in this study are included in the article. Further inquiries can be directed to the corresponding author.

## Abbreviations

ΔCT Gene	delta cycle threshold values
ACQ	Asthma Control Questionnaire
ACT TM	Asthma Control Test TM
ADL	Index of Independence of Daily Living
AMTS	Abbreviated Mental Test Score
AQLQ(S)	The standardized Asthma Quality of Life Questionnaire
BBB	Blood–Brain Barrier
B-H	Benjamini–Hochberg correction
CAT	COPD Assessment Test
CDT	Clock-Drawing Test
CG	control group
CNS	central nervous system
COPD	chronic obstructive lung disease
CREB	cAMP response element binding protein
EDTA	Ethylenediaminetetraacetic Acid
G-CSF	granulocyte colony-stimulating factor
GDS	the 15-item Geriatric Depression Scale
HAM-D	The Hamilton Depression Rating Scale
HIS	Hachinski Ischaemic Score
IADL	The Lawton Instrumental Activities of Daily Living
IgE	Immunglobuline E
IL	Interleukin
MMP	Matrix metalloproteinase
mMRC	modified Medical Research Council dyspnea scale
MMSE	Minimal Mental State Examination
NF-κB	Nuclear factor kappa-light-chain-enhancer of activated B cells
PKA	protein kinase A
ROS	reactive oxygen species
SD	standard deviation
SE	standard error
SF-36	36-item Short Form Healthy Survey
SGRQ-C	The St. George Respiratory Questionnaire dedicated COPD patients
TGF-ß	Transforming Growth Factor ß
TNF-α	Tumor Necrosis Factor α
